# One Health Landscape of Antimicrobial Resistance in Bacteria Isolated from Virginia between 2007–2021

**DOI:** 10.3390/antibiotics13060504

**Published:** 2024-05-29

**Authors:** Jimin Kim, Eunice Ndegwa

**Affiliations:** 1 Columbia College, Columbia University, New York, NY 10027, USA; 2 Agricultural Research Station, Virginia State University, 1 Hayden Drive, Petersburg, VA 23806, USA

**Keywords:** antimicrobial resistance, one health, *E. coli*, *Salmonella*

## Abstract

The emergence of antimicrobial-resistant (AMR) bacteria has become a critical global One Health issue, mainly attributed to the extensive use of antimicrobial agents in human and agricultural settings. Regional and local AMR surveillance data is essential for implementing awareness and mitigation strategies. This article assesses AMR frequency in 1604 bacterial isolates consisting of *Escherichia coli* (*E. coli*) and *Salmonella* spp. isolated from diverse sources in Virginia, including farm animals, wildlife, environment, and food samples from 2007 to 2021. The results are based on the Kirby–Bauer disc diffusion assessment method of susceptibility to select antimicrobial agents, spanning nine distinct categories approved by the US Food and Drug Administration for clinical use. Streptomycin (STR) and tetracycline (TCY) exhibited the highest frequency of resistance in *E. coli* (39.1%) and *Salmonella* (25.2%), respectively. Multidrug resistance (MDR) was evident in 6.6% of *E. coli* and 10.9% of *Salmonella* isolates. Notably, 51% of *E. coli* and 36% of *Salmonella* isolates demonstrated resistance to more than one antimicrobial. None of the tested antimicrobials guaranteed effectiveness against the bacteria isolated from the surveyed sources and regions. The study found heightened MDR and distinct AMR patterns in bacteria isolated from food products compared to other sampled sources. These findings are vital for comprehending the current AMR landscape, prompting the development of strategies to mitigate the emergence of AMR bacteria, and advocating prudent antimicrobial use from a One Health perspective.

## 1. Introduction

The widespread use of antimicrobials in human and agricultural practices has facilitated the extensive dissemination of antimicrobial resistant (AMR) bacteria throughout the environment, presenting a significant global health risk [1]. The U.S. Centers for Disease Control and Prevention (CDC) estimates that over 2.8 million individuals contract infections from AMR bacteria annually, with at least 35,000 fatalities directly attributable to these infections [2]. Responding to the threat, World Health Organization has called for enhanced AMR surveillance [3], and in the US, the FDA has ongoing efforts to bolster antimicrobial resistance surveillance via the National Antimicrobial Resistance Monitoring System (NARMS) [4]. Globally, there are calls for efforts to escalate surveillance, monitoring [5], and discovery [6] of new strategies to combat AMR in bacteria. 

Given the continuous occurrence of foodborne illnesses and other infectious diseases in the United States and the complex nature of AMR transmission pathways, there is a pressing need for continued research on bacterial strains isolated from biological, food, and environmental samples and their potential resistance to antimicrobials. Frequently, foodborne illnesses are excessively treated with antimicrobials, contributing to the proliferation of multidrug resistance (MDR) bacterial strains affecting both humans and animals [7]. Previous studies have also linked this spread of resistance to the use of antimicrobials in veterinary medicine and the transmission of resistant isolates through animal-origin foods to humans [7,8]. Consequently, there was a government call for the reduction of antimicrobial usage in both veterinary and human medicine practices [9] which subsequently resulted in the Veterinary Feed Directive (VFD) that banned use of antibiotics in feed for growth promotion and restricted antibiotic dispensation to only under veterinary supervision. Research by Brown et al. [10] suggests that the primary source of antimicrobials in the general environment is the presence of partially metabolized antimicrobials from humans and animals.

International attention to the risks associated with antimicrobial use in animal production has led to the establishment of various surveillance systems and networks [11]. The U.S. government mandates the monitoring of AMR trends among foodborne bacteria collected from humans, retail meats, and food-producing animals [12]. Additionally, NARMS monitors AMR in foodborne pathogens and tracks the sources and magnitude of AMR in the food supply. Many foodborne illnesses are caused by bacteria, like *E. coli* and *Salmonella* [13]. Each year in the United States, more than 100,000 people go to the hospital and 3000 people die because of foodborne illnesses [14]. Given that *Salmonella* is one of the main bacterial causes of foodborne illness, NARMS particularly monitors this bacterium to assess its resistance to various antimicrobials used in human and veterinary medicine. Conversely, *E. coli* has been related to samples with a lack of or inadequate sanitation [15] and are considered as hygienic indicators [16]. Moreover, the level of AMR in *E. coli* is considered a reliable indicator of the selection pressure exerted by antimicrobial use [17]. Notably, irrespective of the pathogenic status, it is known that AMR elements can also be transmitted between bacteria occupying the same environment [18].

The AMR scourge is a One Health issue necessitating a broad approach to surveillance and monitoring [19]. In the animal industry, the role of companion animals, where antibiotics are used widely, and wildlife in the transmission of AMR elements through the environment remains to be fully studied [20]. Additionally, although the use of antibiotics in aquaculture in most countries is limited to certain classes of antibiotics, it is another animal industry where antibiotics are also widely used, whose status and significance in contribution to AMR is still largely unexplored [21]. 

Antimicrobial resistance poses a worldwide challenge; however, its most substantial and immediate consequences are felt at the local level where the resistant bacteria are identified. Thus, while national surveillance data is important, local data is critical in informing communities on the immediate risk circulating in the environment and clinical settings [22]. Therefore, the current study evaluates the frequency of AMR in *E. coli* and *Salmonella* isolates from a One Health perspective, covering farm animals, wildlife, food, and environmental samples obtained in Virginia between 2007 and 2021.

## 2. Results and Discussion

The frequency of AMR in *E. coli* and *Salmonella* isolates to the 12 antimicrobials tested are summarized in Table 1 and Table 2.

**Figure 1:**
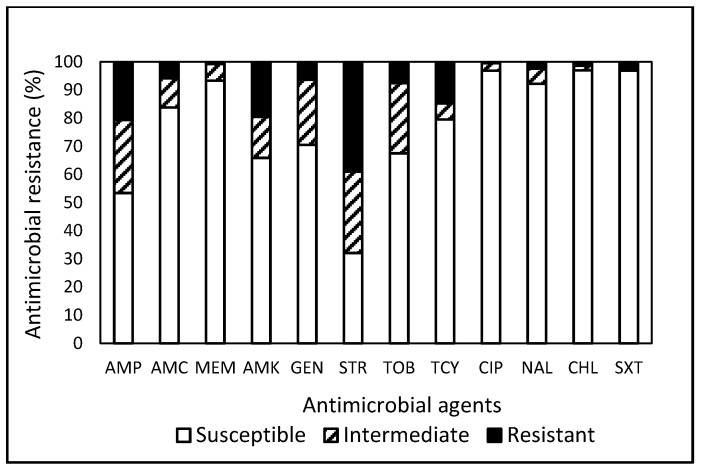
Prevalence of resistance to 12 antimicrobial agents in a total of 1485 *E. coli* isolates obtained from food, water well, ponics, farm, and environmental samples in Central and Eastern Virginia, USA, over the period from 2007 to 2021.

Among the 1485 *E. coli* isolates investigated, 98 (6.6%) exhibited MDR (Table 1). Isolates obtained from the Food Study demonstrated the highest MDR rate at 11.6%, surpassing the MDR rates observed in other studies, including 0% in the Ponics Study, 0.6% in the Farm Study, 3.1% in the Environment Study, and 5.9% in the Water Well Study, indicating that the predominant portion (67 out of 98, 68.4%) was sourced from the Food Study, followed by 16 (16.3%) from the Water Well Study and 14 (14.3%) from the Environment Study. The resistance to streptomycin (STR) was the most common in 580 (39.1%) isolates, followed by ampicillin (AMP, 20.7%), amikacin (AMK, 19.6%), and tetracycline (TCY, 14.9%) (Figure 1). Approximately 23% of the isolates demonstrated susceptibility to the entire array of tested antimicrobials, underscoring that a substantial 77% of the *E. coli* isolates were non-susceptible to at least one antimicrobial agent (Table 1). Although 99.2% and 99.5% of the isolates exhibited susceptibility to meropenem (MEM) and ciprofloxacin (CIP), respectively, none of the tested antimicrobials demonstrated efficacy against the entire spectrum of *E. coli* isolates under evaluation. Additionally, one isolate each obtained from an Italian pork sausage [24] and water well [25] demonstrated non-susceptibility to ten antimicrobials, while one isolate detected in ground pork displayed resistance against eight antimicrobials. Strains isolated from the Food Study exhibited the highest resistance to TCY. In contrast, strains from the Environment, Farm, Ponics, and Water Well Studies displayed the highest resistance to STR, AMP, STR, and STR, respectively, again manifesting distinct AMR patterns in bacteria linked to different sample sources [25,26,27,28,29].

In the global survey conducted by Urban-Chmiel et al. [18], broad distributions of amoxicillin-clavulanic acid (AMC) resistance (ranging from 70.5% to 95%) and TCY resistance (ranging from 50% to 55%) were observed in *E. coli* strains isolated from humans, animals, and the environment. These frequencies were much higher than the resistance levels observed in our study, which showed 5.7% for AMC and 14.9% for TCY. They observed an increased prevalence in community-acquired infections and among isolates from animals. Other studies [30,31] documented instances of CIP-resistant *E. coli* isolated from companion animals, livestock, and humans. Additionally, a report on the 2018 Virginia State and Regional Cumulative Antibiogram [32] indicated that inpatient *E. coli* isolates obtained from Central and Eastern VA exhibited 100% susceptibility to AMK and MEM, with the highest resistance recorded against CIP (24–25%) and AMP (40–50%). However, findings from the current study demonstrated 19.6%, 0.8%, 0.5%, and 20.7% resistances to AMK, MEM, CIP, and AMP, respectively. Discrepancies in resistance patterns between the current study and the state report [32] may be attributed to variations in the antimicrobial panels used, source samples of isolates screened, geographic locations, and the potential development of different degrees of resistance based on environmental exposure, genetic background, and strain type (clinical vs. non-clinical) [33,34]. Incorporating insights from a range of studies [18,25,30,31,32], among the 12 tested antimicrobials, MEM was the only antibiotic reported to be globally effective against *E. coli.*

**Figure 2:**
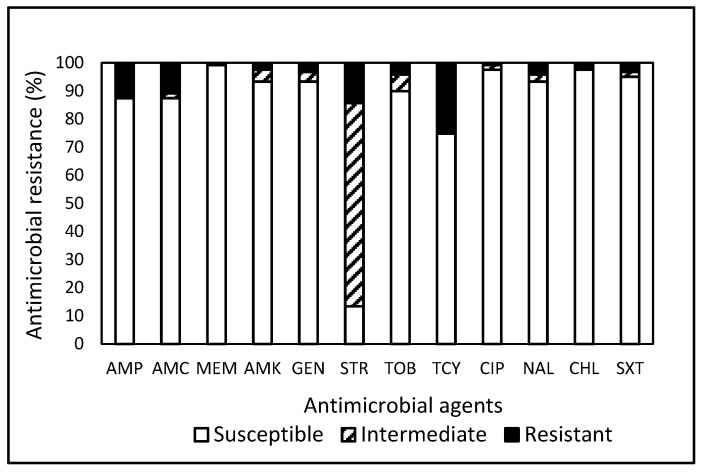
Prevalence of resistance to 12 antimicrobial agents in a total of 119 *Salmonella* isolates obtained from food, farm, and wildlife samples in Central and Eastern Virginia, USA, over the period from 2007 to 2021.

Within the pool of 119 *Salmonella* isolates, 36.1% (43 isolates) exhibited resistance to one or more antimicrobials (Table 2), with 30.3% and 31.3% originating from the Wildlife Study and the Farm Study, respectively. Remarkably, a mere 6.7% of isolates were found to be susceptible to all tested antimicrobials, underscoring that a substantial 93.3% of *Salmonella* isolates were non-susceptible to at least one antimicrobial. In contrast to the current findings, Nesemeier et al. [35] observed higher susceptibility rates (42%) of *Salmonella* isolated from range and feedlot cattle in the North Dakota region of the USA to all antimicrobials tested. As previously mentioned, disparities in resistance patterns can stem from differences in the antimicrobial panels employed, as well as the potential emergence of varying degrees of resistance influenced by environmental exposure and strain types. Table 2 indicates that *Salmonella* spp. isolated from the Food Study exhibited a higher MDR rate at 31.3%, in contrast to the 8.9% MDR rate observed from the Wildlife Study, while none of the *Salmonella* strains isolated from the Farm Study demonstrated MDR. Irrespective of the studies, *Salmonella* isolates exhibited the highest resistance to TCY (Figure 2). Although none of the tested antimicrobials demonstrated efficacy against the entire spectrum of *Salmonella* isolates under evaluation, noteworthy observations included high susceptibilities to MEM (99.2%), CIP (97.5%), chloramphenicol (CHL, 97.5%), AMK (93.3%), GEN (93.3%), and nalidixic acid (NAL, 93.3%) (Figure 2). A significant finding highlighted *Salmonella*, sourced from a gull during the Wildlife Study, as displaying resistance to eight antimicrobials, suggesting wild avians as potential vectors for the dispersion of AMR and pathogenic microbes.

In comparison, a study [36] reported a high prevalence (31.9–40.3%) of MDR with few resistances to GEN (12.5%), STR (18%), and CIP (7.6%) to *Salmonella* isolates obtained from laying hens in North and South America, Africa, Europe, and Asia in 2013–2019. However, no resistance to MEM, CHL, and trimethoprim-sulfamethoxazole (SXT) was detected for these isolates. Additionally, other studies [37,38,39] indicated that *Salmonella* strains obtained from broiler chickens in various geographic regions showed a high level of resistance to NAL (80.3%), AMP (64.8%), STR (33%), SXT (39.3%), and AMC (29.4%) with relatively low resistance to CIP (19%), CHL (13.6%), and GEN (6%). Although susceptibilities of strains isolated in other regions of the USA and European countries to MEM, CIP, CHL, and GEN are about the same level, AMR in *Salmonella* obtained in the USA has generally been higher. Regardless, the observed variability in resistance prevalence among different regions and countries may be attributed to disparities in antimicrobial usage practices, as elucidated by Doyle et al. [34]. Building upon studies [35,40,41,42], the high susceptibility rate (>93%) in *Salmonella* isolates to AMK and MEM underscores the efficacy of these antimicrobials in treating *Salmonella* infections in both veterinary and human medical practices.

Overall, the prevalence of non-susceptibility to the tested antimicrobials was notably high in *E. coli* (76.6%, Table 1) and *Salmonella* (93.2%, Table 2). Contrary resistance patterns (Figure 1 and Figure 2) were observed, with STR in *E. coli* (39.1%) and TCY in *Salmonella* (25.2%). Further analysis revealed that CIP demonstrated the highest efficacy against *E. coli* (96.9%) and MEM against *Salmonella* (99.2%). Approximately 49.6% and 77.9% of the 1604 isolates of *E. coli* and *Salmonella* combined exhibited resistance and non-susceptibility, respectively, to at least one tested antimicrobial. Penicillins and TCY, commonly utilized nationally [43], displayed a relatively high prevalence of resistance in *E. coli* and *Salmonella.* The current analysis of data collected in Central and Eastern VA reveals elevated rates of MDR in *E. coli* and *Salmonella* isolated from food products compared to isolates from other sample sources. *Salmonella* isolates exhibit the highest resistances against TCY, irrespective of sample origin. These findings underscore the pervasive nature and distinct pattern of AMR in bacteria from a One Health perspective.

## 3. Materials and Methods

### 3.1. Bacterial Source

Table 3 outlines sample origins, types, and the corresponding bacteria assessed for AMR frequency presented in this study. Concise details of the sample origins can be found in Section 3.2. Sample origin below. The evaluation encompassed 1604 bacteria isolates, including 1485 *E. coli* and 119 *Salmonella* spp. Isolates were aggregated from diverse research initiatives conducted over a 15-year period (2007–2021) across Central and Eastern Virginia. The *E. coli* isolates comprised 576 sourced from food products acquired from farmers’ markets and local retail markets as part of the ‘Food Study’ [24,44,45,46,47], while 270 were obtained from water well samples (Water Well Study) [25,26], 30 were isolated from aquaponics and hydroponics systems (Ponics Study) [27], and an additional 159 were derived from fecal samples of farm-reared small ruminants (Farm Study) [28]. Furthermore, 450 isolates originated from environmental samples (Environment Study) [29]. The *Salmonella* isolates comprised 16 sourced from the Food Study and 13 obtained from the Farm Study. Additionally, 90 isolates were obtained from fecal samples of wildlife from the Eastern Shore of Virginia (Wildlife Study) [48,49].

### 3.2. Sample Origin

Briefly, for the Food Study, products including fresh fruits, vegetables, and meat were procured from registered farmers’ markets [24,45] and retail stores [46,47] including small independently owned markets and chain supermarkets located in Central Virginia between 2017 and 2020. For the Environment Study [29], feces of livestock and wild avians, water from wastewater treatment plants, and water from a watershed of different land use (crop, forest, pasture, and urban) in Central Virginia between 2020 and 2021 were used. For the Farm Study [28], fecal samples of small ruminants and wild avians were obtained from farms in Virginia between 2007 and 2017. For the Ponics Study [27], samples including fresh produce, produce tank water, fish tank water, biofilter, sludge tank, and fish skin were collected from six schools, three residential sites, and one correctional department between 2019 and 2020. For the Water Well Study [25,26], water samples were obtained from residential water wells in the region between 2016 and 2021. For the Wildlife Study [48,49], fecal material were obtained from deer, geese, ducks, gulls, turtles, and waterfowl in the Eastern Shore of Virginia between 2007 and 2012. Sample preparation followed standard FDA methods [50], ensuring the aseptic process.

### 3.3. Bacterial Isolation and Identification Performed

Bacterial isolation and identification followed AOAC-approved or performance-tested procedures [51]. In brief, the identification of *E. coli* involved culture transfer from lauryl sulfate tryptose broth to EC broth containing 4-methylumbelliferyl-b-D-glucuronide (EC-mug, unless otherwise stated; all media were from Bacto, BD, Sparks, MD. USA), followed by streaking on eosine–methylene blue agar, and confirmation with API 20E test strips (bioMe’rieux, Hazelwood, MO, USA). *Salmonella* identification included the procedures of pre-enrichment in buffered peptone water, enrichment in Rappaport-Vassiliadis broth, and post-enrichment in M broth, streaking on xylose lysine desoxycholate (XLD) agar for isolation and confirmation with API 20E test strips. All confirmed *E. coli* and *Salmonella* isolates obtained above were suspended in Brucella broth containing 20% glycerol, stored at −80 °C, and used for AMR evaluation.

### 3.4. Antimicrobial Resistance (AMR) Evaluation

According to the methodology outlined by Kim et al. [33], antimicrobial susceptibility testing was conducted on Mueller–Hinton agar (MHA) using the Kirby–Bauer disk diffusion method [23]. The confirmed *E. coli* and *Salmonella* isolates were subjected to susceptibility testing against 12 antimicrobial agents that are FDA-approved for clinical use, as outlined in Table 4. Interpretation of antimicrobial susceptibility—categorized as “susceptible”, “intermediate”, and “resistant”—was conducted in adherence to criteria set by the National Committee of Clinical Laboratory Standards [38]. Isolates classified as either resistant or intermediate were collectively termed “non-susceptible”. Additionally, bacteria displaying resistance to at least one antimicrobial agent across three or more categories were classified as multidrug resistant (MDR) [12].

For the AMR evaluation of *E. coli* and *Salmonella*, the resuscitated bacterial suspension in Mueller–Hinton Broth (MHB), which was adjusted to approximately 8 log CFU/mL, was surface plated on Mueller–Hinton Agar (MHA), with subsequent application of antimicrobial discs (Oxoid Ltd., Basingstoke, UK). Incubation for 24 h at 36 °C followed, and inhibition diameter zones were measured in millimeters with a caliper. *E. coli* ATCC 25922 cultured and sub-cultured in MHB served as the control strain for antimicrobial performance assessments. Since the aim of this study was to evaluate the overall trend of AMR emergence in a large number of *E. coli* and *Salmonella* isolates over 15 years from a One Health perspective at the regional level, the AMR prevalence was assessed in a single comprehensive evaluation.

## 4. Conclusions

This article presents the frequency of AMR in bacteria isolated from various sources over fifteen years in Virginia. The findings underscore the pervasive nature of AMR in bacteria within the One Health framework, emphasizing the need for cautious antimicrobial practices in both human and agricultural contexts. The study supplements NARMS data, providing crucial insights for AMR surveillance in Virginia’s food, livestock, wildlife, and water, providing a regional perspective on AMR in *E. coli* and *Salmonella* isolates, with implications at the national level. Ongoing research, encompassing diverse sample sources and bacterial serovars, is essential to unravel factors influencing observed disparities in AMR profiles, deepen our understanding of AMR in bacteria, particularly in environmental contexts, and elucidate genomic relationships among different species, contributing to effective combat strategies.

## 5. Limitations with the Current Study

Limitations in current AMR studies include the absence of data on AMR frequency in bacteria associated with companion animals, despite documented instances. Limited sample availability may also hinder comprehensive representation of the studied area. Nonetheless, these findings offer valuable insights into AMR patterns within a One Health framework. Exploring the role of microbiome and microbiota in bacterial AMR is intriguing, although AMR practices at sampling sites remain unknown. Continued research efforts are crucial to assess correlations between AMR genes and sample sources, shedding light on environmental influences. The novelty of this information underscores the need for further research to identify and mitigate causes of observed AMR profile differences, thereby addressing the emergence and dissemination of AMR bacteria. Sampling bias, particularly in central VA, and the possibility of overrepresentation or underestimation of resistance mechanisms, pose challenges to the generalizability and interpretation of findings. Additionally, without comprehensive data on antimicrobial usage and contextual factors, accurately assessing AMR prevalence and trends remains challenging.

## Figures and Tables

**Table 1:** Prevalence of antimicrobial resistance in 1485 *E. coli* isolates obtained from food, water well, ponics, farm, and environmental samples in Central and Eastern Virginia, USA, over the period from 2007 to 2021 *.

Study (n ^a^)	Nature of AMR ^b^	Number (%) of Isolates Exhibiting Resistance or Non-Susceptibility to Each Quantity of Antimicrobial Agents ^c^										MDR ^d^
		1	2	3	4	5	6	7	8	9	10	
Food (576)	R	94 (16.3)	71 (12.3)	38 (6.6)	13 (2.3)	6 (1.0)	5 (0.9)	4 (0.7)	1 (0.2)	0 (0.0)	0 (0.0)	67 (11.6)
	R+I	122 (21.2)	122 (21.2)	89 (15.5)	61 (10.6)	30 (5.2)	16 (2.8)	12 (2.1)	2 (0.3)	1 (0.2)	1 (0.2)	NA ^e^
Water well (270)	R	40 (14.8)	23 (8.5)	10 (3.7)	8 (3.0)	3 (1.1)	0 (0.0)	0 (0.0)	0 (0.0)	0 (0.0)	0 (0.0)	16 (5.9)
	R+I	51 (18.9)	30 (11.1)	35 (13.0)	18 (6.7)	7 (2.6)	8 (3.0)	7 (2.6)	2 (0.7)	0 (0.0)	1 (0.4)	NA
Ponics (30)	R	1 (3.3)	0 (0.0)	0 (0.0)	0 (0.0)	0 (0.0)	0 (0.0)	0 (0.0)	0 (0.0)	0 (0.0)	0 (0.0)	0 (0.0)
	R+I	8 (26.7)	1 (3.3)	0 (0.0)	0 (0.0)	1 (3.3)	0 (0.0)	0 (0.0)	0 (0.0)	0 (0.0)	0 (0.0)	NA
Farm (159)	R	33 (20.8)	7 (4.4)	0 (0.0)	1 (0.6)	0 (0.0)	0 (0.0)	0 (0.0)	0 (0.0)	0 (0.0)	0 (0.0)	1 (0.6)
	R+I	42 (26.4)	19 (11.9)	2 (1.3)	1 (0.6)	0 (0.0)	0 (0.0)	0 (0.0)	0 (0.0)	0 (0.0)	0 (0.0)	NA
Environment (450)	R	105 (23.3)	151 (33.6)	94 (20.9)	37 (8.2)	4 (0.9)	3 (0.7)	1 (0.2)	0 (0.0)	0 (0.0)	0 (0.0)	14 (3.1)
	R+I	19 (4.2)	21 (4.7)	38 (8.4)	93 (20.7)	137 (30.4)	88 (19.6)	40 (8.9)	9 (2.0)	5 (1.1)	0 (0.0)	NA
Overall (1485)	R	273 (18.4)	252 (17.0)	142 (9.6)	59 (4.0)	13 (0.9)	8 (0.5)	5 (0.3)	1 (0.1)	0 (0.0)	0 (0.0)	98 (6.6)
	R+I	242 (16.3)	193 (13.0)	164 (11.0)	173 (11.6)	175 (11.8)	112 (7.5)	59 (4.0)	13 (0.9)	6 (0.4)	2 (0.1)	NA

* Susceptibility categorization was carried out in accordance with interpretive criteria provided by the National Committee of Clinical Laboratory Standards recommendations [23]; ^a^ number of isolates tested; ^b^ antimicrobial resistance (AMR); R: resistant; I: intermediate; R+I: non-susceptible to antimicrobial agents tested; ^c^ prevalence (%) was presented in resistance and non-susceptibility of isolates to the total number of antimicrobial agents tested [i.e., an isolate exhibiting resistant and intermediate to one and two antimicrobial agents, respectively, was presented under 1 of resistance (R) and 3 of non-susceptibility (R+I).]; ^d^ multi-drug resistance; ^e^ not applicable.

**Table 2:** Prevalence of antimicrobial resistance in 119 *Salmonella* isolates obtained from food, farm, and wildlife samples in Central and Eastern Virginia, USA, over the period from 2007 to 2021 *.

Study (n ^a^)	Nature of AMR ^b^	Number (%) of Isolates Exhibiting Resistance or Non-Susceptibility to Each Quantity of Antimicrobial Agents ^c^								MDR ^d^
		1	2	3	4	5	6	7	8	
Food (16)	R	5 (31.2)	3 (18.8)	1 (6.3)	2 (12.5)	2 (12.5)	0 (0.0)	0 (0.0)	0 (0.0)	5 (31.2)
	R+I	3 (18.8)	4 (25.0)	1 (6.3)	2 (12.5)	2 (12.5)	0 (0.0)	1 (6.3)	1 (6.3)	NA ^e^
Farm (13)	R	1 (7.7)	1 (7.7)	0 (0.0)	0 (0.0)	0 (0.0)	0 (0.0)	0 (0.0)	0 (0.0)	0 (0.0)
	R+I	11 (84.6)	2 (15.4)	0 (0.0)	0 (0.0)	0 (0.0)	0 (0.0)	0 (0.0)	0 (0.0)	NA
Wildlife (90)	R	18 (20.0)	1 (1.1)	4 (4.4)	1 (1.1)	2 (2.2)	1 (1.1)	0 (0.0)	1 (1.1)	8 (8.9)
	R+I	54 (60.0)	17 (18.9)	3 (3.3)	5 (5.6)	3 (3.3)	1 (1.1)	0 (0.0)	1 (1.1)	NA
Overall (119)	R	24 (20.2)	5 (4.2)	5 (4.2)	3 (2.5)	4 (3.4)	1 (0.8)	0 (0.0)	1 (0.8)	13 (10.9)
	R+I	68 (57.1)	23 (19.3)	4 (3.4)	7 (5.9)	5 (4.2)	1 (0.8)	1 (0.8)	2 (1.7)	NA

* Susceptibility categorization was carried out in accordance with interpretive criteria provided by the National Committee of Clinical Laboratory Standards recommendations [23]; ^a^ number of isolates tested; ^b^ antimicrobial resistance (AMR); R: resistant; I: intermediate; R+I: non-susceptible to antimicrobial agents tested; ^c^ prevalence (%) was presented in resistance and non-susceptibility of isolates to the total number of antimicrobial agents tested [i.e., an isolate exhibiting resistant and intermediate to one and two antimicrobial agents, respectively, was presented under 1 of resistance (R) and 3 of non- susceptibility (R+I).]; ^d^ multi-drug resistance; ^e^ not applicable.

**Table 3:** Summary of the study type, sample origin, sample type, and the corresponding bacteria evaluated.

Study Type	Sample Origin	Sample Type	Bacteria Species (n ^a^)	
			*E. coli*	*Salmonella* spp.
Food	Farmers’ markets and retail stores	Fruits, vegetables, and meat	576	16
Environment	Livestock, wild avian, wastewater treatment plant, and watershed	Feces and water	450	NA
Farm	Small ruminants	Feces	159	13
Ponics	Aquaponics and hydroponics system	Vegetables, water, biofilter, sludge, and fish skin	30	NA
Water well	Well	Water	270	NA
Wildlife	Deer, ducks, geese, gulls, and turtles	Feces	NA ^b^	90
Total number of isolates			1485	119

^a^ number of isolates evaluated; ^b^ not applicable.

**Table 4:** A list of antimicrobials and interpretive criteria used [23,45] *.

Antimicrobial Category	Antimicrobial Agent and Its Abbreviation	Concentration (µg/Disk)	Zone Diameter (mm)		
			S	I	R
Penicillins	Ampicillin (AMP)	10	>17	14–16	<13
β-lactamase inhibitor combinations	Amoxicillin-clavulanic acid (AMC)	30	>18	14–17	<13
Carbapenems	Meropenem (MEM)	10	>23	20–22	<19
Aminoglycosides	Amikacin (AMK)	30	>17	15–16	<14
	Gentamicin (GEN)	10	>15	13–14	<12
	Streptomycin (STR)	10	>15	12–14	<11
	Tobramycin (TOB)	10	>15	13–14	<12
Tetracyclines	Tetracycline (TCY)	30	>15	12–14	<11
Fluoroquinolones	Ciprofloxacin (CIP)	5	>21	16–20	<15
Quinolones	Nalidixic acid (NAL)	30	>19	14–18	<13
Phenicols	Chloramphenicol (CHL)	30	>18	13–17	<12
Folate pathway inhibitors	Trimethoprim-sulfamethoxazole (SXT)	25	>16	11–15	<10

* Interpretive criteria: S, susceptible; I, intermediate; R, resistant to antimicrobial agents tested.

## Data Availability

Given the limited availability of sample origin and identical sample types assessed, prevalence of AMR in bacteria may only partially represent some samples associated with tested bacterial species within the study area. It is important to note that any mention of trade names or commercial products in this publication serves the sole purpose of providing specific information and does not imply any recommendation or endorsement by Virginia State University. The authors wish to clarify that this work does not, nor was it intended to, suggest the superiority or inferiority of the mentioned commodities and antimicrobials compared to others. There is no implied endorsement or criticism of the safety or efficacy of these products.
